# Intronic *PRRT2* mutation generates novel splice acceptor site and causes paroxysmal kinesigenic dyskinesia with infantile convulsions (PKD/IC) in a three generation family

**DOI:** 10.1186/s12881-016-0281-7

**Published:** 2016-03-03

**Authors:** Axel Weber, Jonas Kreth, Ulrich Müller

**Affiliations:** Institut für Humangenetik, Justus-Liebig-Universität Gießen, Gießen, Germany; Abteilung für Neuropädiatrie, Klinik für Kinder und Jugendliche, Klinikum Leverkusen gGmbH, Leverkusen, Germany

**Keywords:** Paroxysmal kinesigenic dyskinesia (PKD), Infantile convulsions (IC), PKD/IC, Dystonia 10, PRRT2, Novel splice acceptor site

## Abstract

**Background:**

Mutations in *PRRT2* cause autosomal dominant paroxysmal kinesigenic dyskinesia with infantile convulsions (PKD/IC).

**Case presentation:**

A previously not recognized intronic *PRRT2* mutation (c.880-35G > A; p.S294Lfs*29) was found in an 18 month old girl with IC and in her mother with classical presentation of PKD. The mutation results in a novel splice acceptor site in intron 2 of *PRRT2*. Due to frameshift and a subsequent premature stop-codon the resulting transcript appears to render the PRRT2 protein non/dysfunctional and is the likely cause of disease in this family.

**Conclusion:**

Our findings expand the mutational spectrum of this disease.

## Background

Benign infantile afebrile convulsions and kinesigenic dystonia are the hallmarks of paroxysmal kinesigenic dyskinesia with infantile convulsions (PKD/IC, dystonia 10; OMIM #128200, #605751 and #602066). Onset of convulsions (IC) is commonly during the first year of life. Kinesigenic involuntary movements (PKD) develop somewhat later in early childhood. While convulsions usually resolve by 2 years of age, abnormal movements occur throughout life. PKD manifests as brief (less than a minute) but frequent (up to 100/day) dystonic and choreatiform attacks that are precipitated by abrupt movements.

PKD/IC is inherited as an autosomal dominant trait with high penetrance. The disease locus was assigned to the short arm of chromosome 16 and a positional cloning approach has identified *PRRT2* as the underlying disease gene [1–3] *PRRT2* encodes “proline-rich transmembrane protein 2” and appears to be required for normal synapse function.

*PRRT2* mutations identified in PKD/IC to date include frameshift, missense and splice site mutations and most commonly a C insertion (c.649-650insC) (reviewed in [4]). Furthermore microdeletions at chromosome 16p11.2 are known to cause PKD/IC [5, 6]. Known splice site mutations affect the well established splice donor (GT) and splice acceptor (AG) sites. Here we describe an intronic *PRRT2* mutation in PKD/IC that generates a novel splice acceptor site which alters the transcript.

## Case presentation

The proposita was born after 37 weeks of an uneventful pregnancy to non-consanguineous parents. She weighed 2500 g (15^th^ percentile), her length was 45 cm (3^rd^ to 15^th^ percentile) and her head circumference 33 cm (25^th^ to 50^th^ percentile). First afebrile convulsions occurred at age 6 months. They lasted up to 3 min and resolved spontaneously. Convulsions were mainly generalized, did not start focally, and were followed by muscular atonia and cyanosis. Muscle tone was normal between attacks. Cerebrospinal fluid analysis, auditory screening, EEG and cranial MRI were normal. Inborn errors of metabolism were excluded. She does not display developmental delay. No convulsions occurred during medication with oxcarbacepine (daily dose: 10 mg per kilogram of body weight).

The proposita’s mother is affected by early-onset generalized paroxysmal dystonia. She was born in 1977, developed normally during childhood and did not have afebrile benign or febrile convulsions. At nine years of age she developed dystonic episodes triggered by sudden passive movements, stress, and fright. The involuntary movements lasted for up to 20 s. There was no infringement of consciousness during episodes. The attacks occurred daily. Muscle tone was normal between attacks. Medication with oxcarbacepine (daily dose: 300 mg) abolished attacks entirely.

Although the maternal grandfather could not be examined, he is reported to have suffered from episodic dyskinesias as well, similar to the mother’s episodes in terms of age of onset, appearance and duration. Based on the mother’s statement no additional family members are known to have neurological symptoms.

## Materials and methods

DNA was isolated from EDTA blood by standard methods (QIAGEN DNA-Blood and Tissue Kit). RNA was isolated from PAX-Blood using the RNeasy mini Kit of QIAGEN. Reverse transcription was performed following a standard protocol using SuperScript-III reverse transcriptase (Thermofisher) and random hexamer primers (Thermofisher). Dideoxy sequencing was performed according to standard procedures. Primers for amplification and sequencing of the genomic sequence of *PRRT2* exons and adjacent intronic sequences were published previously [5]. Primers spanning exons 2 and 3 (RT-PCR) were:

- upper: 5′-CCCATGTGGCCTGTCAAC-3′
- lower: 5′-CGCCTAAGTTGATGACGCA-3′

Multiple alignment of 100 vertebrate species and measurements of evolutionary conservation of guanine at position was performed applying the UCSC Vertebrate Multiz Alignment & Conservation tool at (http://genome.ucsc.edu/index.html). The multiple alignments were generated using multiz and other tools available through the UCSC/Penn State Bioinformatics comparative genomics alignment pipeline.

## Results and discussion

**Fig. 1:**
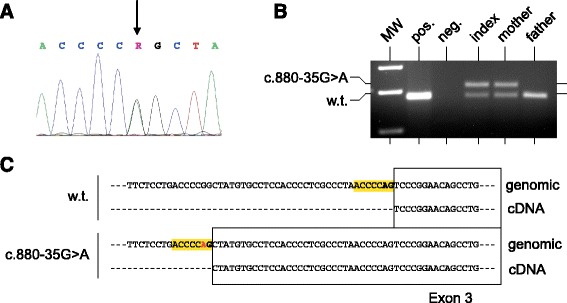
**a** Electropherogram of intron 2/exon3 sequence of *PRRT2* in the patient. Note the G > A transition (arrow) c.880-35G > A. **b** Different sized wild-type (=179 bp) and mutant (=211 bp) PCR products on an ethidium bromide stained gel. Lane 1: molecular weight standard (MW), Lane 2: positive control DNA (human placental), Lane 3: negative control (water), Lane 4–6: DNA of the family members investigated. **c** Novel splice acceptor site due to c.880-35G > A and altered sequence of exon 3 of the PRRT2 transcript

DNA sequencing in the proposita and her mother revealed a G-A transition in intron 2 of *PRRT2* (Fig. 1). This base change is located 35 base pairs upstream of the wild-type splice acceptor site (AG) of exon 3 [hg19: chr16:29825620G > A (*PRRT2* c.880-35G > A; p.S294Lfs*29)] (Fig. 1). G at this position is evolutionarily highly conserved and identical in almost all vertebrates tested. No variant found generated a novel consensus AG-splice acceptor site. Furthermore, the G-A change is not listed in public databases (NCBI, DGV, dbSNP). Substitution of G by A at this position is predicted to result in the generation of a novel consensus splice acceptor site (AG). The Alternative Splice Site Predictor (ASSP) [7] assigned a score of 8.170 (confidence 0.456) to the new splice acceptor site. This was even higher than the score of 4.192 (confidence 0.330) calculated for the wild-type site.

In order to test the prediction of a functional new splice acceptor site we performed RT-PCR analysis using RNA extracted from the patient’s and her mother’s lymphocytes. As shown in figure, mRNAs of two sizes were found. One corresponds to the wild-type transcript, the second one is extended by 36 bases thus confirming that it was generated by splicing of exon 2 to the novel splice acceptor site in intron 2. Fig. also shows the one band found in unaffected persons, such as in a control and in the patient’s unaffected father (Fig. 1). Splicing to this novel site results in a frameshift and premature termination of the amino acid chain.

Although the proposita and her mother carried the identical mutation, the patients’ phenotype differed. While the proposita displayed the classical picture of IC, followed by PKD, the mother and her father had PKD only. Different phenotypes in the presence of identical mutations of “disease genes” are a common finding in most monogenic disorders probably owing to modifier genes and environmental factors. In the case of *PRRT2* mutations an entire spectrum of diseases has been described in addition to PKD/IC. These diseases include familial hemiplegic migraine, benign paroxysmal torticollis, episodic ataxia and paroxysmal non-kinesigenic dyskinesias [8]. Unlike the observations in the present family, it might be that at least some of these highly variable phenotypes are also associated with mutations in specific regions of *PRRT2* in addition to modifier genes and environmental factors.

## Conclusion

In conclusion we have described a novel mutation in *PRRT2* that results in a larger transcript of the gene. Abnormal function of the predicted altered PRRT2 protein appears to cause PKD/IC probably by a dominant negative effect.

## Consent

The family described in this report has provided us with their written consent to have their clinical histories published.

## Abbreviations

PRRT2: proline-rich transmembrane protein 2
PKD: paroxysmal kinesigenic dyskinesia
IC: infantile convulsions
DNA: deoxyribonucleic acid
RNA: ribonucleic acid
RT-PCR: reverse transcriptase polymerase chain reaction
NCBI: National Center for Biotechnology Information
DGV: database of genomic variants
dbSNP: database of single nucleotide polymorphisms
ASSP: alternative splice site predictor
